# Magnetic Carbon Microspheres as a Reusable Adsorbent for Sulfonamide Removal from Water

**DOI:** 10.1186/s11671-017-2295-2

**Published:** 2017-09-06

**Authors:** Kewei Dai, Fenghe Wang, Wei Jiang, Yajun Chen, Jing Mao, Jian Bao

**Affiliations:** 1 Jiangsu Province Key Laboratory of Environmental Engineering, School of Environment, Nanjing Normal University, Nanjing, 210023 China; 2 Jiangsu Provincial Key Laboratory of Materials Cycling and Pollution Control, School of Geography Science, Nanjing Normal University, Nanjing, 210023 China; 3 National Special Superfine Powder Engineering Research Center, Nanjing University of Science and Technology, Nanjing, 210094 China; 4 Jiangsu Provincial Academy of Environmental Science, Nanjing, 210036 China

**Keywords:** Magnetic Carbon Microspheres (MCMs), Adsorption, Sulfonamide, Reusability

## Abstract

Novel reusable magnetic carbon microspheres (MCMs) were prepared by hydrothermal method with glucose as carbon source and Fe_3_O_4_ nanoparticles as magnetic raw materials. And adsorption performance of MCMs for sulfonamide removal from water was investigated in detail. The results indicated that the calcination temperature and calcination time had significant effects on the surface area and its volume porous of MCMs. When MCMs were calcined in 600 °C for 1 h, the surface area and volume porous of MCMs were 1228 m^2^/g and 0.448 m^3^/g, respectively. The adsorption results showed that the adsorption data fitted well with the Langmuir isotherm model and followed pseudo-second-order kinetics. When the pH value was changed from 4.0 to 10.0, the adsorption capacity of MCMs for sulfonamide was decreased from 24.6 to 19.2 mg/g. The adsorption capacity of as-synthesized MCMs achieved 18.31 mg/g after it was reused four times, which exhibited a desirable adsorption capacity and reusability.

## Background

Pharmaceutical antibiotics are widely used in the world to treat diseases and improve the growth rate of animals. However, it has been found that antibiotics have serious adverse effects on the aquatic environment, which has attracted growing concern in recent years [1–3]. Among all the antibiotics, sulfonamide antibiotics are usually heavily used in clinical, animal husbandry and aquaculture. They are very stable and poorly absorbed in the digestive tract with only a small portion of sulfonamide antibiotics metabolized or absorbed. When they are discharged into the environment, sulfonamide antibiotics have been frequently detected in wastewater treatment plants, groundwater, surface water, soil, sediments, etc. [4–6]. The sulfonamide antibiotic residues can not only damage the environment but also pose a significant risk to human health. Therefore, it is necessary to investigate new technology to effectively removing these antibiotic residues from water.

Carbon materials have drawn much attention for their chemical inertness, biocompatibilities, and thermal stabilities [7–9]and have been investigated extensively in the field of separation, catalyst, and adsorption [10–12]. However, the traditional carbon materials are difficult to separate from solution when they are used as adsorbent. The conventional methods are mainly filtration and centrifugation, which are inconvenient and low efficient especially when the working condition is complicated. With the development of nanotechnology in recent years, carbon materials combined with nano-magnetic materials, namely magnetic carbon microspheres (MCMs), have been paid much more attention which can be easily separated using a magnet. These magnetic carbon composites have been used as adsorbents for pollutant removal from water, such as methyl blue [13] and phenol and nitrobenzene [14]. Zhu et al. have reviewed the synthesis and application of magnetic carbon composites [15].

In this paper, we introduced a novel approach for synthesizing new magnetic carbon microspheres (MCMs) with high surface area by hydrothermal method, in which glucose and Fe_3_O_4_ nanoparticles were used as raw materials. And adsorption performance of MCMs for sulfonamide removal from water was evaluated in detail.

## Methods

### Chemicals and Materials

FeCl_3_·6H_2_O, ethanol, ethylene glycol, and sulfonamide were purchased from Sinopharm Chemical Reagent Co., Ltd. NaAc·3H_2_O, glucose, KCl, NaOH, and HCl were from Nanjing Chemical Reagent Co., Ltd. ZnCl_2_ was purchased from Xilong Chemical Co., Ltd. Distilled water was used in all the experiments.

### Preparation of Fe_3_O_4_ Nanoparticles

Fe_3_O_4_ nanoparticles were prepared via the hydrothermal method as reported in [16]. FeCl_3_·6H_2_O (1.35 g) and NaAc·3H_2_O (3.60 g) were dissolved in 40 mL of ethylene glycol to form a homogeneous solution and then was translated to a teflon-lined stainless autoclave (100 mL capacity), heated to 200 °C for 8 h. After it was cooled to room temperature, the resulting product was washed with deionized water and ethanol for three times, respectively.

### Preparation of MCMs

0.1 g Fe_3_O_4_ nanoparticles, appropriate dosage of glucose, and 60 mL distilled water were added into a 100-mL beaker and then were stirred to make the Fe_3_O_4_ nanoparticles homodispersed. The solution was poured into a 100 mL autoclave and heated to 200 °C for 11 h. The MCMs obtained were washed twice with deionized water and ethanol.

MCMs were immersed in the 40% ZnCl_2_ solution [17, 18] and then were dried in a vacuum drying oven. The as-synthesized MCMs were putted in a tuber furnace and heated under nitrogen atmosphere. Thus, the calcinated and activated MCMs were obtained. The resulting MCMs were washed, used 50 mL deionized water five to eight times till the concentration of Zn^2+^ was less than 0.05 mg/L, and the MCMs were dried in a vacuum drying oven for sulfonamide adsorption.

### Characterization

MCMs were characterized using transmission electron microscopy (TEM, Model Tecnai 12, Philips Co., Ltd., Holland) and field emission scanning electron microscopy (FE-SEM, Model S-4800, Hitachi Co., Ltd., Japan). Magnetic properties of the MCMs were measured at room temperature using a vibrating sample magnetometer (VSM, Model 7410, Lake Shore Co., Ltd., USA). Nitrogen adsorption and desorption performance were performed using a specific surface area analyzer (Model Coulter SA3100, Beckman Co., Ltd., USA). The surface areas were calculated using the Brunauer–Emmett–Teller (BET) equation. Surface zeta potential was measured by a zeta potential analyzer (ZS90, Malvern Instruments, UK).

### Adsorption Procedure

The adsorption experiments were carried out in 50-mL conical flasks in a temperature-controlled orbital shaker (QHZ-98A, Taicang Bio-Instrument Manufacture Co., Ltd). To reduce the photodegradation possibility of sulfonamide, all the conical flasks contained sulfonamide solutions and appropriate dosage of MCMs were enclosed with aluminum foil and shaken at room temperature (300 K) in 120 rpm. After adsorption was finished, MCMs were separated from sulfonamide solution by magnet. The concentration of sulfonamide was measured at 258 nm by an ultraviolet–visible spectrophotometer (UV–vis, Model 759S, China), and adsorption capacity of MCMs (*Q*_*e*_, mg/g) was calculated according to Eq. (1):

1 $$ {Q}_e=\frac{\left({C}_0-{C}_e\right)\times V}{m} $$

where *Q*_*e*_ is the adsorption capacity at equilibrium (mg/g); *C*_*0*_ and *C*_*e*_ denote the initial and equilibrium concentrations of sulfonamide (mg/L), respectively; *V* is the volume of sulfonamide solution (50 mL); *m* is the mass of the adsorbent MCMs (mg).

### The Reusability Method of MCMs

To assess the reusability of MCMs, 1 g/L MCMs was added in 25 mg/L sulfanilamide solution in which its concentration of pharmaceutical plant drainage was simulated. The adsorption capacity of MCMs was calculated when the adsorption reached equilibrium. The absorbed MCM could be separated and dispersed in distilled water and desorbed by adding 0.1 mol/L NaOH until the pH value reached 10.0, then ultrasounded at 500 W for 10 min, and this process was repeated three times [19, 20]. Then, MCMs were washed by distilled water repeatedly till the pH = 7. In all the experiments, the magnet was employed to separate MCMs from aqueous solution.

## Results and Discussion

### TEM of MCMs

**Fig. 1:**
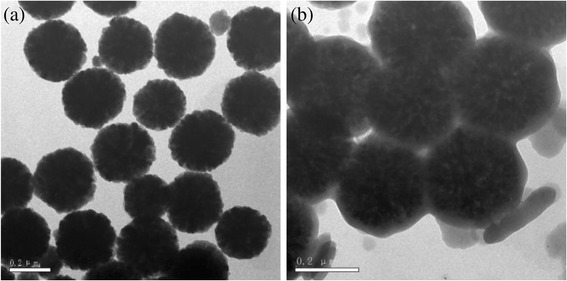
TEM images. **a** Fe_3_O_4_ nano/microspheres. **b** MCMs

The TEMs of Fe_3_O_4_ nano/microspheres and MCMs were showed in Fig. 1.

As shown in Fig. 1a, the sizes of Fe_3_O_4_ nano/microspheres were around 200 nm and dispersed uniformly. After Fe_3_O_4_ nano/microspheres were reacted with glucose by hydrothermal method, the carbon was covered on the surface of Fe_3_O_4_ microspheres (Fig. 1b). At the same time, some carbon microspheres were formed which was in accordance with previous works of Cakan et al. [21].

### FT-IR and XRD Spectrum of MCMs

**Fig. 2:**
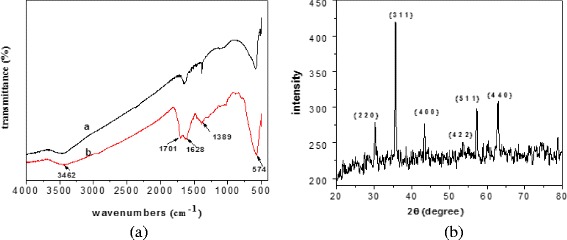
FT-IR and XRD spectrum of the resulting products. **a** FT-IR spectrum (a: Fe_3_O_4_, b: MCMs). **b** XRD spectrum of  Fe_3_O_4_

The FT-IR and XRD spectrum of the resulting products Fe_3_O_4_ nano/microspheres and MCMs were showed in Fig. 2.

The resulting Fe_3_O_4_ and MCMs had adsorption peak near 574 cm^−1^, which was the characteristic peak for Fe_3_O_4_. There was a wide absorption peak near 3462 cm^−1^ for Fe_3_O_4_ and MCMs, which suggested the resulting Fe_3_O_4_ and MCMs had –OH functional group. The peaks in 1701 and 1621 cm^−1^ were vibration absorption of carbonyl and alkene, which attributed to carbonization of glucose in hydrothermal method.

It is found that all the reflection peaks can be assigned to the diffraction from (2 2 0), (3 1 1), (4 0 0), (4 2 2), (5 1 1), and (4 4 0) crystal planes of cubic structure of Fe_3_O_4_ (JCPDS no. 19-0629), which indicated the formation of magnetite nanoparticles [22].

### Surface Area and Porous Volume of MCMs

**Fig. 3:**
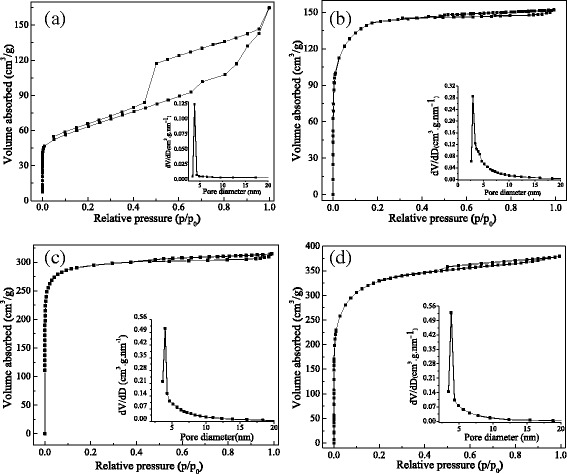
Nitrogen adsorption–desorption isotherms and the pore size distributions of MCMs. **a** MCMs without ZnCl_2_ impregnation. **b** MCMs calcined at 550 °C for 1 h without ZnCl_2_ impregnation. **c** MCMs with ZnCl_2_ impregnation for 1 h were calcined at 550 °C for 1 h. **d** MCMs with ZnCl_2_ impregnation for 1 h were calcined at 600 °C for 1 h

The N_2_ adsorption–desorption isotherms and their relevant Brunauer–Emmett–Teller (BET) pore size distribution of the MCMs prepared were showed in Fig. 3, and their parameters of BET surface area (*S*_BET_), pore volume, and pore size were listed in Table 1.

**Table 1:** The parameters of MCMs obtained at different reaction conditions

MCMs	Temperature (°C)	Time (h)	*S*_BET_ (m^2^/g)	Pore volume (cm^3^/g)	Pore size (nm)
a	–	–	223	0.082	3.7
b	550	1	356	0.175	2.9
c	Impregnation 1 h, 550	1	1028	0.423	3.8
d	Impregnation 1 h, 600	1	1228	0.445	3.6

As shown in Fig. 3a, the adsorption curve of MCMs without ZnCl_2_ impregnation and calcination belonged to the II-type adsorption isotherm, which usually presented non-porous material; the hysteresis loop could be assigned to type H2 according to the IUPAC nomenclature, which implied that the porous volume was formed by packing of grains [23]. At the same time, the sample in Fig. 3a possessed low surface area (223 m^2^/g) and low porous volume (0.082 m^2^/g), although it had a large porous size (3.7 nm), which confirmed the porosity were the packing porosity but not the primary porosity.

The MCM samples in Fig. 3b–d were all calcined at high temperature and had similar adsorption isotherms. As shown in Fig. 3, all the isotherm curves increased rapidly at low relative pressure, which implied there were micropores in the MCM samples and facilitated to strong adsorption; while at high relative pressure, these curves exhibited a plateau, which demonstrated that no further adsorption took place, and these curves were the typical I-type adsorption isotherm. In the adsorption–desorption procedure, there appeared hysteresis loop at the high relative pressure. The phenomenon suggested the presence of micropores or mesopores and the hysteresis loop could be classified into type H4. This type hysteresis loop usually appears on activated carbon [24]. The corresponding pore size distribution data calculated by the BJH method showed that the pore size mainly distributed below 3–5 nm, which just confirmed that large amounts of mesopores exist on the surface of the MCMs. Although all the three MCM samples had the similar adsorption isotherm as activated carbon, their surface areas and porous volumes were different, as listed in Table 1.

Compared with the MCM samples (c) and (d), MCM samples (b) had much lower surface area (356 m^2^/g) and porous volume (0.175 cm^3^/g), which were calcined at 550 °C for 1 h without ZnCl_2_ impregnation. Therefore, it can be concluded that ZnCl_2_ played a vital role in increasing the surface area and porous volume. As previously reported, ZnCl_2_ is used for activate agents in preparation process of activated carbon and can result in degradation of cellulosic material and dehydration, which can cause charm and aromatization of the carbon skeleton and creation of the pore structure [25]. Furthermore, the MCM samples (c) and (d) were all impregnated 1 h, and the only one difference was their calcination temperature, which led to the surface area and porous volume being changed significantly. The higher the temperature, the greater the surface area and porous volume. So the MCM samples (d) were chosen for the following magnetic properties and adsorption research because of its highest surface area and porous volume.

### Magnetic Properties of MCMs

**Fig. 4:**
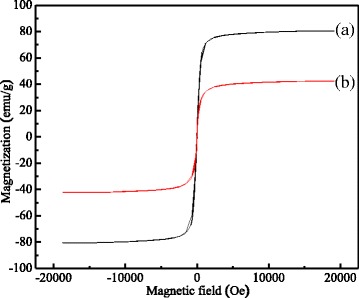
Magnetic properties of the MCMs. (**a**) The saturation magnetization of the pure Fe_3_O_4_ nano/microspheres. (**b**) The saturation magnetization of MCMs

The magnetic properties of the MCMs were investigated using a vibrating sample magnetometer (VSM), and its hysteresis loop of Fe_3_O_4_ nano/microspheres (a) and MCMs calcined at temperature 600 °C for 1 h (b) was showed in Fig. 4 which was measured at room temperature (300 K) with VSM.

As shown in Fig. 4, the saturation magnetization of the pure Fe_3_O_4_ nano/microspheres was 80.3 emu/g, which was smaller than 92.0 emu/g, the saturation magnetization of bulk Fe_3_O_4_ [19], while its saturation magnetization of MCMs was 42.3 emu/g, which was much less than that of pure Fe_3_O_4_ nano/microspheres and bulk Fe_3_O_4_. This sharp decrease indicated there was much carbon adhered to the surface of Fe_3_O_4_ nano/microspheres. However, the magnetic cores in MCMs possessed high saturation magnetization, the carbon which adhered to the surface of Fe_3_O_4_ nano/microspheres almost had no effects on their magnetic responsibility. Their remnant magnetization and coercivity were found to be zero, indicating Fe_3_O_4_ nano/microspheres and MCMs were superparamagnetic, which implied that the MCMs could be controlled and separated by using applied magnetic field.

### Adsorption Isotherm

Langmuir and Freundlich equations were commonly used in adsorption equilibrium for illustrating the adsorption interaction, which were listed in Eqs. (2) and (3) [26, 27]:

2 $$ \frac{C_{\mathrm{e}}}{Q_{\mathrm{e}}}=\frac{1}{Q_{\mathrm{m}}{K}_L}+\frac{C_{\mathrm{e}}}{Q_{\mathrm{m}}} $$

3 $$ \ln {Q}_{\mathrm{e}}=\ln {K}_F+\frac{1}{\mathrm{n}}\ln {C}_{\mathrm{e}} $$

where *C*_e_ (mg/L) is the equilibrium concentration of sulfonamide, *Q*_e_ (mg/g) is the sulfonamide amount adsorbed per gram of adsorbent MCMs under equilibrium, *Q*_m_ (mg/g) is the theoretical maximum adsorption capacity of MCMs for sulfonamide, and *K*_L_ (L/mg) is the constant depicting the affinity in the process of Langmuir adsorption; where *K*_F_ is the Freundlich empirical constants indicative of the relative adsorption capacity of the MCMs, and 1/n is the constant indicative of the intensity of the Freundlich adsorption [27].

**Fig. 5:**
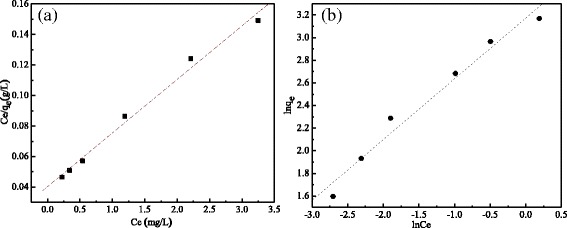
The adsorption isotherms of MCMs. **a** Langumir model. **b** Freundlich model

The Langumir and Freundlich adsorption isotherms were showed in Fig. 5, and their characteristic adsorption parameters are listed in Table 2.

**Table 2:** The relevant parameters of the two models

Model	*K*	*Q*_m_ (or n)	*R* ^2^
Langumir	*K*_L_ = 3.264	*Q*_m_ = 27.8551	0.9923
Freundlich	*K*_F_ = 3.0564	*n* = 2.099	0.9882

As shown in Fig. 5 and Table 2, there was a linear relationship in the Langumir and Freundlich isotherm models and had no big difference in the two models. In the Langumir model, the theoretical maximum adsorption capacity of MCMs for sulfonamide was *Q*_m_ = 27.8551 mg/g. In the Freundlich model, the values of the constants *K*_F_ and 1/n were calculated to be 3.0564 L/g and 0.476, respectively. Since the value of 1/n is less than 1, it indicated a favorable adsorption. As far as the linear coefficient value (*R* ^2^) was concerned, compared with the Freundlich model, the linear coefficient value (*R* ^2^) of the Langmuir isotherm model was greater than the other one, which indicated that the equilibrium adsorption data fitted the Langmuir isotherm better.

### Adsorption Kinetics

To provide some insight of the adsorption process and their relationship between the MCMs and sulfonamide and further clarify the adsorption type as well as the influencing factors, two kinetic models, the pseudo-first-order equation and the pseudo-second-order equation, were used to study the adsorption kinetics of MCMs, which were given by Eqs. (4) and (5) [28–30]:

4 $$ \ln \left({Q}_e-{Q}_t\right)=\ln {Q}_e-{K}_1t $$

5 $$ \frac{t}{Q_t}=\frac{1}{K_2\times {Q_e}^2}+\frac{t}{Q_e} $$

where *Q*_*e*_ and *Q*_*t*_ denoted the adsorption capacity of sulfonamide at the equilibrium state and at time of *t*; *K*_1_ (min^−1^) and *K*_2_ (g mg^−1^ min^−1^) are the modulus of pseudo-first-order and pseudo-second-order adsorption, respectively. The linear plot of ln(*Q*_*e*_ *− Q*_*t*_) versus *t* gave the slope of *− K*_1_ and an intercept of ln*Q*_*e*_. A plot of (*t/Q*_*t*_) versus *t* gave a slope of (*1/Q*_*e*_) and intercept of 1/(*K*_*2*_ × *Q*_*e*_^2^).

The kinetic curves and the calculated parameters of the models with their linear coefficient (*R* ^2^) are listed in Table 3.

**Table 3:** The kinetic curves and the calculated parameters of the adsorption kinetic models

Model	Pseudo-first-order	Pseudo-second-order
Eq.	*Y* = − 0.147*X* + 2.622	*Y* = 0.039*X* + 0.085
*K*	*K*_1_ = 0.147	*K*_2_ = 0.0179
*Q*_e_	13.8 (mg/g)	25.65 (mg/g)
*R* ^2^	0.972	0.993

As shown in this Table 3, the correlation coefficient in the pseudo-second-order equation was more than that of the pseudo-first-order model and showed good linearity, which indicated that the adsorption of sulfonamide by MCMs was likely kinetically controlled as a second-order reaction rather than a first-order one, and the adsorption rate-limiting step may include chemisorption.

### Effects of pH Values on MCMs’ Adsorption Capacity

**Fig. 6:**
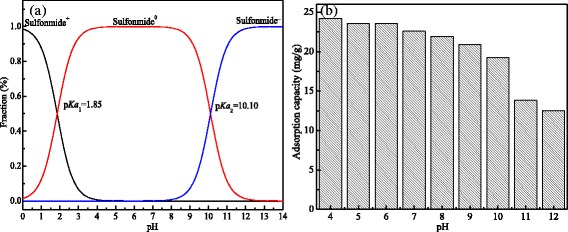
Effects of pH on the sulfanilamide speciation and adsorption capacity of sulfonamide. **a** Sulfanilamide speciation varied with pH. **b** Adsorption capacity of MCMs varied with pH

The natural pH value of 25 mg/L sulfonamide solution was found to be 6.0. The pH value was changed from 4.0 to 10.0 by adjusting with 0.1 mol/L NaOH and 0.1 mol/L HCl. The effects of pH values on the adsorption capacity of MCMs were investigated, and the results are showed in Fig. 6.

MCMs’ adsorption capacity was influenced by the sulfanilamide speciation and the charges in the surface of MCMs. As shown in Fig. 6, the adsorption capacity of MCMs was reduced from 24.22 to 12.48 mg/g when the pH was increased from 4 to 12. The higher adsorption performance in acid solution than in alkaline solution may be related to the pKa of sulfonamide and zero point potential of MCMs. When the pH was in the range of 4.0~6.0 which was in the acidic condition, its zeta potential was 2.96 mV, the surface of MCMs was mainly positive charge, and the sulfanilamide existed mainly by natural molecular state (sulfonamide^0^) at the same time [28, 29]. Thus, sulfonamide could be easily absorbed onto MCMs, which indicated MCMs had a higher removal efficiency than those in alkaline condition; while in the alkaline condition, its zeta potential was − 4.01 mV, the positive charge on the surface of the MCMs was modified into negative charge, and the sulfanilamide speciation was varied into negative ones (sulfonamide^−^), which led to the electrostatic repulsion effect between sulfonamide species and MCMs because of their same kind of charges. Furthermore, sulfanilamide was easily dissolved in the alkaline solution [30], which made it having more tendency to dissolve in the solution rather than be absorbed to the MCMs. Therefore, the adsorption capacity was decreased significantly, which implied that MCMs can be desorbed effectively in alkaline solution, such as pH = 10.

### Effects of Temperature and Ion Strength on MCMs’ Adsorption Capacity

The effects of temperature and ion strength (KCl as the ion regulator) on MCMs’ adsorption capacity were investigated, and the results are listed in Table 4.

**Table 4:** Effects of temperature and ion strength on MCMs’ adsorption capacity

Item (°C)	0.01 mmol/L	0.05 mmol/L	0.005 mmol/L
10	20.98	22.83	25.17
20	18.66	19.72	23.26
30	16.52	17.63	19.87

As shown in Table 4, with increasing of temperature and the ion strength, its adsorption capacity of MCMs decreased, which may be attributed to the adsorption competition of KCl with sulfonamide. Adsorption capacity comparison of MCMs with other absorbents used for sulfonamide from aqueous solutions is listed in Table 5.

**Table 5:** Adsorption capacity comparison of MCMs with other absorbents reported for sulfonamide

Adsorbent	Sulfonamide	*Q*_m_ (mg/g)	Reference
TCPP/Fe_3_O_4_-GO	2 mg/L	13.9	[31]
Activated carbons	17.2–172 g/L	31	[32]
Fe_3_O_4_ nanoparticles	25 mg/L	10.83	This study
MCMs	25 mg/L	24.22	This study
Highly crosslinked polystyrene (HCPS)	3.4 mg/L	8.6	[33]

This comparison suggests that MCMs can serve as an alternative absorbate in removing sulfonamide. At the same adsorption conditions, the adsorption capacity of resulting MCMs and Fe_3_O_4_ nanoparticles was 24.22 and 10.83 mg/g, respectively, which means its adsorption capacity of MCMs mainly comes from carbon.

### The Reusability of MCMs

**Fig. 7:**
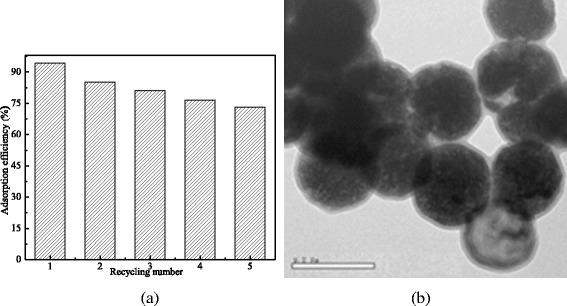
The reuse frequencies of the MCMs and its morphology after reused four times. **a** The reuse frequency. **b** The morphology of the MCMs after reused four times

The reuse frequency of the MCMs is displayed in Fig. 7a, and its morphology and microstructures of the MCMs after reused four times are shown in Fig. 7b.

As shown in Fig. 7, the adsorption removal efficiency was 94.28%, and its adsorption capacity for sulfonamide was calculated to be 23.6 mg/g. After being washed with diluted NaOH solution and reused, its adsorption efficiency was decreased with the recycling number increasing. The obtained adsorption removal efficiencies were 85.23, 81.17, 76.53, and 73.23% for the second-, third-, fourth-, and fifth-time adsorption, respectively, which were corresponding to 21.31, 20.29, 19.13, and 18.31 mg/g of adsorption capacity. Comparing Fig. 7b with Fig. 1b, morphology and microstructures of the MCMs after reused four times were not changed. Consequently, MCMs could be reused for sulfanilamide removal.

## Conclusions

The MCMs possessing sensitive magnetic responsibility and high surface area were successfully synthesized by a facile hydrothermal method, and its specific surface area and pore volume reached up to 1228 m^2^/g and 0.445 m^3^/g, respectively. The adsorption of sulfanilamide by MCMs fitted well with the Langmuir isotherm model and followed pseudo-second-order kinetics. After being desorbed with NaOH solution, the adsorbent of MCMs could be recycled. The main findings of the present work will contribute to designing and synthesizing novel absorbents, and a better understanding of their adsorption physicochemical processes.
